# Cognitive Effects of Montelukast: A Pharmaco-EEG Study

**DOI:** 10.3390/brainsci11050547

**Published:** 2021-04-27

**Authors:** Fabian Schwimmbeck, Wolfgang Staffen, Christopher Höhn, Fabio Rossini, Nora Renz, Markus Lobendanz, Peter Reichenpfader, Bernhard Iglseder, Ludwig Aigner, Eugen Trinka, Yvonne Höller

**Affiliations:** 1 Department of Neurology, Christian Doppler University Hospital, Paracelsus Medical University, 5020 Salzburg, Austria; f.schwimmbeck@salk.at (F.S.); w.staffen@salk.at (W.S.); f.rossini@salk.at (F.R.); Nora.Renz@pmu.ac.at (N.R.); e.trinka@salk.at (E.T.); 2 Centre for Cognitive Neuroscience (CCNS), Department of Psychology, University of Salzburg, 5020 Salzburg, Austria; s1022428@stud.sbg.ac.at; 3 Spinal Cord Injury and Tissue Regeneration Center Salzburg (SCI-TReCS), Paracelsus Medical University, 5020 Salzburg, Austria; ludwig.aigner@pmu.ac.at; 4 Neuroscience Institute, Christian Doppler University Hospital, 5020 Salzburg, Austria; 5 Laboratory for Sleep, Cognition and Consciousness Research, Department of Psychology, University of Salzburg, 5020 Salzburg, Austria; 6 Medical Practice for Pulmonology Lobendanz, 5020 Salzburg, Austria; lungen@lobendanz.at; 7 Medical Practice for Pulmonology Reichenpfader, 5020 Salzburg, Austria; peter.reichenpfader@medway.at; 8 Department of Geriatric Medicine, Christian Doppler University Hospital, Paracelsus Medical University, 5020 Salzburg, Austria; b.iglseder@salk.at; 9 Institute of Molecular Regenerative Medicine, Paracelsus Medical University, 5020 Salzburg, Austria; 10 Karl Landsteiner Institute for Neurorehabilitation and Space Neurology, 5020 Salzburg, Austria; 11 Faculty of Psychology, University of Akureyri, 600 Akureyri, Iceland

**Keywords:** montelukast, EEG, cognitive tests, anti-inflammatory drug, leukotriene receptor antagonist

## Abstract

Montelukast is a well-established antiasthmatic drug with little side effects. It is a leukotriene receptor antagonist and recent research suggests cognitive benefits from its anti-inflammatory actions on the central nervous system. However, changes in brain activity were not directly shown so far in humans. This study aims to document changes in brain activity that are associated with cognitive improvement during treatment with Montelukast. We recorded EEG and conducted neuropsychological tests in 12 asthma-patients aged 38–73 years before and after 8 weeks of treatment with Montelukast. We found no significant changes on neuropsychological scales for memory, attention, and mood. In the EEG, we found decreased entropy at follow up during rest (*p* < 0.005). During episodic memory acquisition we found decreased entropy (*p* < 0.01) and acceleration of the background rhythm (*p* < 0.05). During visual attention performance, we detected an increase in gamma power (*p* < 0.005) and slowing of the background rhythm (*p* < 0.05). The study is limited by its small sample size, young age and absence of baseline cognitive impairment of the participants. Unspecific changes in brain activity were not accompanied by cognitive improvement. Future studies should examine elderly patients with cognitive impairment in a double-blind study with longer-term treatment by Montelukast.

## 1. Introduction

The aged brain faces various structural, functional and biochemical changes that increase the vulnerability to neurodegenerative diseases and cerebrovascular disorders resulting in cognitive decline, behavioral disturbances and seizures [1,2,3,4]. The prevalence of dementia in 65-year-olds is 6.4% and increases exponentially with progressing age [5]. Additionally, normal ageing is associated with multiple cognitive deteriorations such as impairments in memory, attention, or executive cognitive functions [6,7]. With the number of people over 65 set to double to 1.5 billion by 2050 [8], finding treatments that alleviate age-related symptoms is vitally important.

There is increasing evidence demonstrating that patients with asthma suffer of reduced cognitive functions [9]. Moreover, a nationwide cohort study in Taiwan demonstrated that the risk of dementia development was significantly increased in patients with asthma in comparison to the general [10]. Obviously, this urges the question whether effective asthma treatments might improve cognitive functions in patients with asthma, or whether asthma treatments might lower the risk of developing dementias.

Montelukast is an approved antiasthmatic drug that has recently gained attraction due to its beneficial effects on brain structure and function. It is a leukotriene receptor antagonist initially designed to block the cysteinyl receptor 1 (CysLT1R) in the lungs to prevent inflammation-induced asthmatic bronchoconstriction, but more recent data illustrated that it blocks also other leukotriene receptors such as GPR17. Importantly, leukotriene receptors are expressed throughout the body including the CNS, particularly on microvessel cells, microglia, astrocytes, neurons, and stem and progenitor cells [11], where they mediate certain detrimental effects of leukotrienes such as neuronal cell death, neuroinflammation, blood–brain barrier (BBB) disruptions, and impairment of stem and progenitor functions. The production of leukotrienes is elevated in the aged brain and in brain with neurodegenerative diseases with microglia being the main local production site [12]. In addition, leukotrienes that are produced outside the brain, for example in immune cells, can penetrate the BBB and exert their functions in the brain. Thus, it is reasonable to hypothesize whether blocking leukotrienes through montelukast might counteract inflammation and other detrimental processes that are associated with neurodegenerative diseases, and with cognitive decline in the brain [13]. Indeed, [13] demonstrated that a 6 weeks treatment of Montelukast rejuvenated the brain of aged rats (20 months). Thereby, Montelukast improved learning and memory functions, accompanied by reduced neuroinflammation, a restored BBB integrity and enhanced neurogenesis [13]. In subsequent studies, these outcomes have further been successfully translated to an animal model of Dementia with Lewy Bodies and of Alzheimer’s disease (AD), where treatment with montelukast restored memory, reduced the alpha-synuclein load, and impaired neuroinflammation [14,15].

These findings suggest a pleiotropic efficacy of montelukast that, when translated to humans, may open new avenues to alleviate age-related cognitive decline and to treat neurodegenerative diseases such as dementia with Lewy bodies or Alzheimer’s dementia.

To date, there is limited evidence for a similar effect of montelukast on the human CNS, but initial results are promising. A retrospective analysis based on data from the Norwegian Prescription Database showed a small neuroprotective effect: Patients prescribed montelukast for asthma treatment had a lower risk of developing dementia than patients taking other antiasthmatics [16]. A follow-up study that included a database on neuropsychological test scores found improved cognitive and neurological functioning associated with prior montelukast use [17]. These results and the potential cognitive and psychiatric side effects suggest that montelukast has effects on the CNS. However, no controlled clinical study directly demonstrated potential effects on the CNS of montelukast in humans.

In this explorative study, we investigate whether 8 weeks treatment with montelukast has an effect on cognition and electrophysiological brain activity in patients with asthma bronchiale. We aimed to address this question using neuropsychological tests and quantitative analysis of EEG recordings conducted during rest and cognitive stimulation.

At the cognitive level, we expected to measure the effects of montelukast through improved memory performance in line with previous findings in rodents [13,14]. In addition, we assessed possible adverse events and screened for potential effects on various neuropsychological modalities, such as attention, inhibition control, mood, and psychiatric symptoms.

At the functional level, a large body of literature has shown the ability of pharmaco-EEG to detect neural changes induced by drugs [18,19]. Furthermore, the effects of montelukast on the EEG was recently demonstrated in an animal model of epilepsy, where montelukast acted as an anticonvulsant by reducing epileptiform activity in the EEG [20], [21]. In addition, treatment with montelukast in a mouse model of stroke restored the reduced spectral power in the local field potential recorded in ischemic brain tissue to baseline [22]. Consequently, we hypothesized that quantitative analyses of the human EEG might reveal possible effects of montelukast. We, therefore, extracted several EEG features, such as spectral power, spectrum weighted mean frequency, Hjorth parameters, or entropy, that have already been shown to be sensitive to alterations associated with ageing, cognitive decline, Alzheimer’s dementia, or general pathology [23,24,25,26,27]. In particular, a slowing of the dominant rhythm in the background EEG [23,25], an increase of delta, and a decrease in alpha and beta power has been associated with ageing [28] and is even progressed in AD [29]. Additionally, the reduction of complexity in the EEG signal has been reported in both aged individuals and in patients with AD [30,31,32]. Given the possible effect of montelukast, we expected a reversed pattern due to the treatment. That is, a general shift of power to higher frequency domains, an elevation of the mean frequency and an increase in complexity as measured by Shannon’s entropy.

## 2. Materials and Methods

### 2.1. Participants

Asthma patients who had started monotherapy for the first time with montelukast with the sole indication to treat their asthma independent of this study were recruited by outpatient pulmonologists and voluntarily participated in this study. The inclusion criteria were a minimum duration of 8 weeks of treatment with montelukast and age between 18 and 80 years. In addition, our cognitive tests required good German language skills. Following the guidelines for pharmaco-EEG studies [18], potential candidates with excessive, regular consumption of alcohol, (more than 3 units/day for women or 4 units/day for men; 1 unit is 1/4 L of wine or 1/2 L of beer) tobacco (more than 5 cigarettes per day), or caffeine (more than 4 cups per day), a rapidly progressive neurological disorder, a psychiatric history, or substance abuse were excluded. For women of childbearing age, a negative pregnancy test was required for inclusion. The severity of asthma was classified according to the guidelines of [33] into four categories: intermittent, mild persistent, moderate persistent, or severe persistent asthma.

### 2.2. Ethics

Ethical approval (415-E/2044/15-2017) for this study was obtained from the ethics committee in Salzburg, Austria. The study was conducted in accordance with the Declaration of Helsinki and registered as a clinical trial (EudraCT Number: 2016-003061-25). All participants gave their written informed consent before they participated in the study.

### 2.3. Study Design and Testing Procedure

**Figure 1:**
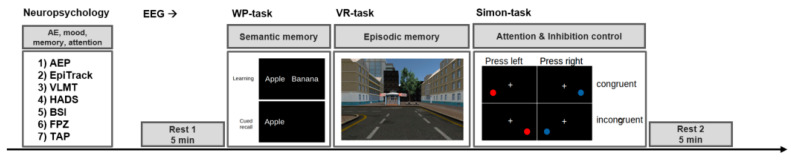
Examination procedure. The examination included a neuropsychological assessment and an EEG recording. During the neuropsychological assessment, the patients were screened for adverse events, mood and psychiatric symptoms, memory, attention, and executive functions. Next, the EEG recordings were conducted starting with a 5 min resting period (Rest 1), followed by three cognitive tasks (semantic memory, episodic memory, attention, and inhibition control), succeeded by a final 5 min resting period (Rest 2). This procedure was conducted for the baseline examination before the medication started, and the same procedure was repeated after 8 weeks treatment with montelukast (using parallel versions of the cognitive tasks). Abbreviations: AE = adverse event, AEP = adverse event profile, VLMT = verbal learning and memory test, HADS = hospital anxiety and depression scale, BSI = Barratt impulsiveness scale, FPZ Fragebogen zur Persönlichkeit bei zerebralen Erkrankungen (clinical personality scales), TAP Thematischer Apperzeptionstest (attention), WP = word-pair learning task, VR = virtual reality-task.

This clinical trial was designed as an unblinded, non-randomized within-subject pilot study. That is, for each patient, a baseline testing (BASELINE condition) was performed before the treatment started, and the same testing procedure was repeated after 8 weeks of medication (MONTELUKAST condition). The test procedure included a neuropsychological examination followed by and an EEG recording. The EEG recording consisted of three parts. First, we conducted a 5-min resting-state EEG for which participants were instructed to relax with their eyes open (Rest 1). Next, participants had to perform three cognitive tasks (Figure 1). Finally, a second 5-min resting-state EEG was recorded to account for possible changes due to cognitive stimulation (Rest 2). For each cognitive task and for each neuropsychological measure, we used parallel versions for the follow-up examination.

### 2.4. Cognitive Tasks during the EEG

Virtual reality task: To examine episodic memory performance changes, we implemented a virtual reality task programmed in Unity 3D [34]. In the learning part of this task, participants were asked to navigate through a virtual town and to remember as many details as possible (taking about 4 min). The recall-part of this test includes a recall and a recognition task. The recall task requires the participants to spell out remembered details, which are noted on a structured grid of responses [34,35]. Two different towns were used for the first and second testing.

Word-pair task: Aiming to assess an effect on semantic memory performance, we implemented an emotional verbal-memory task using the software Presentation^®^ (Neurobehavioral Systems, Inc., Berkeley, CA, USA). In the learning part of this task, participants were presented a set of 60 pairs of German nouns. For each word pair, participants had to indicate whether the pair is related or unrelated in order to control for learning strategy. The recall part consists of a cued recall with the presentation of the first word only. The stimulus material was balanced by psycholinguistic algorithms concerning word frequency, length, and valence, by using the Berlin affective word list [36]. As such, there are each 20 negative, neutral, and positive word pairs, balanced for relatedness and unrelatedness.

Simon task: To measure possible changes in attention and inhibition control, we implemented the Simon task using presentation. The Simon effect is the observation that reaction time tends to be slower when a target cue is presented in the visual hemisphere contralateral to the execution of a motor response to that cue (e.g., keypress) [37,38]. We implemented visual form of the Simon task, in which patients are asked to respond as quickly as possible to the color of a cue presented on a computer screen. In our case, patients had to press the “A” key with their left index finger in response to red dots and with the right index finger the “L” key in response to blue dots. The task consisted of 400 trials divided by 3 breaks into blocks of 100 trials. Each block of trials consisted of 60% congruent trials (cue on the same side as the response key) and 40% incongruent trials (cue on the contralateral side) presented in randomized order. In each trial, patients had to focus a fixation cross until the target cue (red or blue dot) appeared for 50 ms on either the left or right side of the screen. The onset latency of the target cue was randomized and ranged from 1500 to 2000 ms. The patients were instructed to respond as quickly and as accurately as possible.

### 2.5. Electrophysiological Recordings

The EEG was recorded using a 32-channel 16-bit BrainAmp MR amplifier (BrainProducts, Inc., Gilching, Germany) and a 29 Ag–AgCl-electrodes (Fp1, Fp2, F3, F4, C3, C4, P3, P4, O1, O2, F7, F8, T7, T8, P7, P8, Fz, Cz, Pz, FC1, FC2, CP1, CP2, FC5, FC6, CP5, CP6, TP9, and TP10) scalp EEG cap (Easycap, Herrsching-Breitbrunn, Germany). An additional EOG electrode was mounted under the right eye in order to control for eye movement artefacts. The signal was online referenced against FCz and digitized at a sampling rate of 2500 Hz. Impedances were kept under 8 kΩ. All recordings were performed using the Brain Vision Recorder software (BrainProducts, Inc. Gilching, Germany). Technical problems during the first recording session required exclusion of the complete EEG data of patients 1 and 2 from the analysis, reducing the sample size from 12 to 10. Missing data for the cognitive parts in two patients during session 2 led to a further reduction of data in the cognitive EEG part (*n* = 8).

### 2.6. Signal Preprocessing

In order to prepare the data for further analysis pipelines implemented in Matlab (R2020a, The Mathworks, Massachusetts, USA), we performed basic preprocessing (filtering, downsampling, and artefact marking) with the software Brainvision Analyser 2.0 (BrainProducts, Inc., Gilching, Germany). The signal was bandpass filtered between 0.5 and 100 Hz using a digital Butterworth zero-phase filter (time constant 0.3 s, 48 dB/oct), and an additional 50 Hz notch filter was applied to attenuate line noise. Subsequently, the data was downsampled to 256 Hz. To correct eye-movement artefacts, we carried out a semiautomatic ICA algorithm. Muscle and other artefacts were detected in an automatic approach according to the following criteria: A maximum gradient of 50 µV/ms, a maximal allowed absolute difference of 200 µV during an interval of 200 ms and a lowest allowed absolute difference during an interval of 100 ms was 0.5 µV. The result of this artefact detection was reviewed visually and further processed using a custom script implemented in Matlab to retain the optimum of artefact-free data. In a first step, channels that were visually identified as broken or noisy were interpolated over the whole signal length by averaging the signal of neighboring electrodes using the ft_channelrepair function implemented in the fieldtrip toolbox [39]. As TP9 and TP10 were highly affected by muscle artefacts in almost all patients, we decided to exclude these electrodes from further analysis. Subsequently, we interpolated bad intervals of artefacts involving less than three channels. Then, all remaining intervals containing artefacts were excluded from the data. This procedure was performed for each resting- and task-condition. Remaining trials or data-epochs with a duration smaller than 1 s were discarded. Finally, the data was re-referenced against the common average and segmented in 1 s epochs.

### 2.7. Quantitative EEG Features

The pioneering nature of this study demanded to analyze the EEG data in an exploratory approach. Based on a literature research on alterations of the EEG in the context of ageing, dementia, and general pathology, we selected several features in the time domain and frequency domain that have the potential to be sensitive to the possible effects of Montelukast medication. In the following, we briefly describe the selected measures, which we all calculated on the level of the 1 s data epochs:

Hjorth parameters: Hjorth introduced activity, mobility, and complexity as EEG features specifically intended to parameterize clinical EEG traces [40]. The activity is defined as the variance of the time series, also denoted as the mean power. The mobility is the square root of the slope of the signal normalized by the variance, which can be seen as mean frequency. Complexity is the fourth statistical moment of the power spectrum and is an estimate for the deviance of the signal from an ideal sine curve, which can be interpreted as bandwidth, i.e., change in frequency. The Hjorth parameters can be calculated as follows:

$$A=var\left(x\left(t\right)\right),\;M=\sqrt{\frac{var\left(\frac{dx\left(t\right)}{dt}\right)}{var\left(x\left(t\right)\right)}},\;C=\frac{M\left(\frac{dx\left(t\right)}{dt}\right)}{M\left(x\left(t\right)\right)},\;$$

where x(t) is the time-series.

Hjorth parameters have been used in various clinical studies, e.g., for seizure prediction [26] and classification of the EEG of Alzheimer’s dementia [41]. For the latter, a reduction in mobility and increase in complexity has been reported for patients with AD [41]. We calculated these features in stationary mode with the implementation in the BioSig Matlab toolbox [42].

Brain-rate: Brain-rate or spectrum-weighted EEG frequency was proposed as a quantitative indicator of mental arousal and a potential measure for the clinical EEG classification [43]. Brain-rate is an estimator of the weighted mean frequency in the bandwidth from 2 to 18 Hz and is calculated as follows:

$$f_{b}=\underset{i}{\sum }f_{\left(i\right)}\frac{p_{i}}{P}$$

with the predefined set of frequency bands f = [2, 4, 6, 10, 18 Hz], where $p_{i}$ is the corresponding mean power and $P=\overset{\;}{\underset{i}{\sum }}p_{i}.\;$Brain-rate has been calculated using the implementation in the BioSig toolbox [42].

Shannon entropy: Entropy is a measure of uncertainty in a stochastic process, e.g., an EEG time series. We calculated entropy for the time domain with the following equation:

$$H\left(X\right)=-\overset{n}{\underset{i}{\sum }}p\left(x_{i}\right)log_{2}p\left(x_{i}\right)$$

where *n* is the number of bins, *x* is the series of binned data, and $p\left(x_{i}\right)$ is the probability of observing value $x_{i}$ (see also [44]). We estimated the bin size by applying the Friedmann Diaconis rule for each EEG epoch. For the comparability of the entropy values, we took the grand average of all estimates, resulting in 15 bins and used this value for all entropy calculations. As entropy is proportional to the maximal information content of a signal, it is hypothesized that reduced complexity is an indicator of pathology or ageing [30,31,32]. Consequently, we expected an elevated entropy due to montelukast medication.

Spectral Power: Power spectra were obtained by applying the fast Fourier transform on demeaned 1 s data epochs that were tapered with a Hanning window to avoid spectral leakage. The frequency range of interest was 0.5–45 Hz. For the calculation, we used the ft_freqanalyis function implemented in the fieldtrip toolbox [39]. For further statistical comparisons, the power spectra were normalized by the mean power of the frequency range from 0.5 to 45 Hz.

### 2.8. Neuropsychology

For the neuropsychological examination, a battery of standardized tests was used to cover the broad spectrum of possible cognitive effects, impacts on mood, and adverse events: We performed the following tests at baseline and follow-up:

- German version of the adverse events profile of [45]; translation was performed in 2004 by Hoppe/Helmstaedter. This instrument was used in order to systematically record adverse events.
- EpiTrack [46] was chosen in order to have a rapid assessment of various cognitive functions. EpiTrack includes the following subscales:
  - Interference: measures response inhibition;
  - Connecting numbers: measures visuo-motor speed;
  - Connecting numbers and letters: measures mental flexibility;
  - Maze test: measures visuo-motor anticipation;
  - Verbal fluency: measures rapid lexical access;
  - Inverted digit span: measures working memory.
- Verbal learning and memory test [47] was used to measure verbal memory.
- Hospital anxiety and depression scale [48] was used to measure mood and anxiety.
- Barratt impulsiveness scale [49] was used to measure impulsiveness and aggression.
- Clinical personality scales (FPZ) [50] was used to measure psychiatric effects.
- TAP (Thematischer Apperzeptionstest) [51] was used as a test for attentional performance.

### 2.9. Statistics

As stated before, we conceptualized this pilot study to measure within-subject effects in response to treatment with montelukast. Within this framework, we performed group-level analyses comparing the first examination before the treatment started (BASELINE) vs. a second examination after 8 weeks of medication (MONTELUKAST). Due to the small sample size, our analysis entirely relied on non-parametric statistics. The significance level was set to 0.05 and adjusted for multiple comparisons when necessary.

For each EEG feature and each EEG-condition (REST 1, REST 2, WP LEARNING, WP RECALL, VR LEARNING, and SIMON TASK), we averaged the obtained data over epochs or trials. For the Simon task, we additionally subdivided into congruent and incongruent trials. For each (univariate) feature, this resulted in a 1 × 27 vector (feature-value × electrodes) except for power, for which we obtained a 40 × 27 matrix (power per frequency bin × electrodes). Then, we conducted cluster-based permutation tests based on the T-statistic for dependent samples for each feature vector within each EEG-condition. To this end, datapoints were partitioned across the two conditions BASELINE × MONTELUKAST such that in both partitions there were datapoints from BASELINE and MONTELUKAST. The *t*-test performed on these shuffled partitions yields a result under random conditions. Performing this procedure a large number of times allows one to estimate the likelihood of significant test results under random conditions and thus comparing the actual test result to the distribution of random results. In contrast to a randomization test, for this permutation test, all possible unique permutations were performed, which is $2^{n}$. This resulted in 1024 permutations for each contrast within the resting EEG conditions (*n* = 10) and 256 permutations for each contrast performed within the cognitive EEG conditions (*n* = 8).

For all behavioral measures, that is, count of recalled memory items (WP and VR-task), mean reaction time and accuracy (Simon-task), and the neuropsychological tests, we performed Wilcoxon signed-rank tests based on the raw values of each measure or subscale. *p*-values were corrected for multiple comparisons with the Benjamini–Hochberg algorithm. All statistical calculations were performed in Matlab using the fieldtrip toolbox [39] for permutation-testing and the built-in function of the Wilcoxon signed-rank test.

## 3. Results

### 3.1. Patient Demographics

Thirteen asthma patients (nine women, one dropout), aged 38–73 years participated in the experiments. Twelve participants (Mdn 57.5 years, nine women, all right-handers) completed the trial. The diagnoses of the study participants ranged from intermittent to moderate persistent asthma bronchiale and included unclassifiable asthma-COPD overlap syndromes. All patients were treated with 10 mg/kg/day montelukast with asthma treatment as indication, independent of this trial. The mean duration of medication from baseline to follow-up examination was 61.8 days (SE = 6.5 days). One patient dropped-out after the first examination due to personal reasons. No adverse events or psychiatric side effects were observed.

### 3.2. Behavioral Within-Subject Contrasts

#### 3.2.1. Neuropsychology

All the behavioral results are presented in Table 1. In summary, there was no effect of montelukast on any neuropsychological function. For memory, attention, and executive functions, this contradicted our hypothesis.

#### 3.2.2. Cognitive Tasks during the EEG

**Figure 2:**
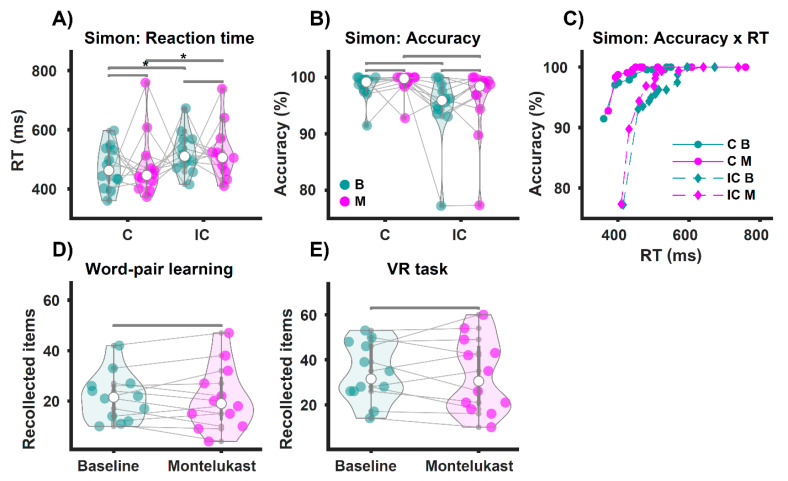
Behavioral results of the cognitive tests during the EEG. * *p* < 0.05. (**A**) Shows the reaction times for congruent (**C**) and incongruent (IC) trials of the Simon task. Participants were slower on the IC trials than on the C trials (Simon effect), both at baseline (B, green) and on the montelukast trials (M, purple). However, we observed no difference in response times when comparing baseline vs. montelukast for C and IC trials. In (**B**), accuracy indicates correct responses per condition as a percentage. We found no differences in accuracy for each comparison. (**C**) Shows accuracy as a function of response time. In the bottom panel, (**D**,**E**) show the results for participants’ performance on the memory tasks. There was no difference in the number of recalled word pairs for the semantic memory task (word pair learning, (**D**)), comparing baseline vs. montelukast. We also found no difference in the number of recalled items for the episodic memory task (navigation through a virtual town, (**E**)).

For the Simon task, we replicated the well-established Simon effect, i.e., a significant enhancement of reaction time for incongruent trials compared to congruent trials [37,38]. Which is a proof of concept of our implementation of the Simon task. This effect was found for both, baseline (C Mdn = 462 ms), (IC Mdn = 510 ms), *z* = 3.06, *p* = 0.049, *r* = 0.62 and the follow-up testing (C Mdn = 446 ms), (IC Mdn = 506 ms), *z* = 2.98, *p* = 0.049, *r* = 0.61 (Figure 2A). The Simon effect was not reflected in the accuracy, as our tests revealed no difference within both baseline (C Mdn = 99%), (IC Mdn = 96%), *z* = −2.5, *p* = 0.106, *r* = −0.51, and follow-up (C Mdn = 100%), (IC Mdn = 98%), *z* = −2.67, *p* = 0.087, *r* = −0.54 (Figure 2B).

Next, we analyzed the behavioral data of the Simon task across baseline vs. follow-up examinations., We did not find any difference for the reaction times comparing congruent trials (B Mdn = 462 ms), (M Mdn = 446 ms), *z* = −0.31, *p* = 0.801, *r* = −0.06, and incongruent trials (B Mdn = 510 ms), (M Mdn = 506 ms), *z* = −0.39, *p* = 0.801, *r* = −0.08; neither did we find any difference for accuracy comparing within congruent trials (B Mdn = 99%), (M Mdn = 100%), *z* = −1.48, *p* = 0.404, *r* = 0.3 and within incongruent trials (B Mdn = 96%), (M Mdn = 98%), *z* = 1.26, *p* = 0.419, *r* = 0.26.

For the semantic memory task (WP-Task), there was no difference between the number of recalled items for baseline (Mdn = 21.5) compared to the montelukast treatment (Mdn = 19), *z* = 0.31, *p* = 1, *r* = 0.06 (Figure 2D). We also found no difference in episodic memory performance (VR-Task) contrasting the number of recollected items for BASELINE (Mdn = 31.5) with MONTELUKAST (Mdn = 30.5), *z* = 0.59, *p* = 1, *r* = 0.12 (Figure 2E).

All in all, there was no effect of montelukast treatment on episodic memory, semantic memory or attention as measured by the tasks conducted during EEG recordings.

### 3.3. Within-Subject Cluster-Based Permutation Tests for EEG Features

For readability, we only reported the significant results here; all other results can be seen in Table S1. As this analysis was exploratory with no a priori assumptions, this analysis can only give limited interpretable insights into specific brain-region and specific frequencies of the effect [52].

**Figure 3:**
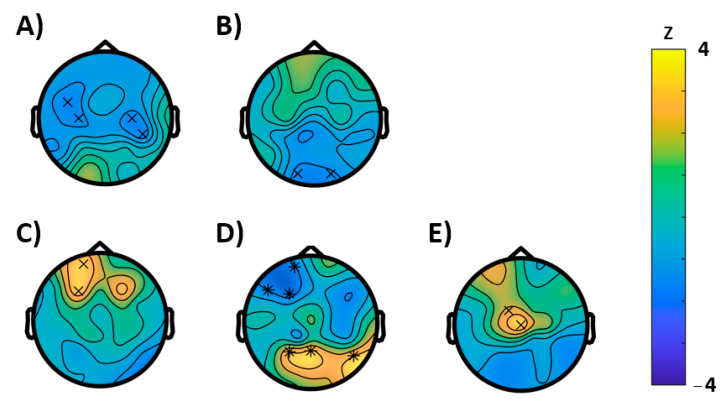
Results of the cluster based permutation tests (BASELINE vs. MONTELUKAST, cluster corrected, x = *p* < 0.05, * = *p* < 0.01). (**A**) Rest 1, entropy; (**B**) WP-task recall, Hjorth activity; (**C**–**E**) VR-task: (**C**) brain-rate, (**D**) entropy, and (**E**) Hjorth activity.

In the resting-state EEG, we only found significant differences between baseline and montelukast examination for the rest 2 condition and here solely for the entropy. Following [16,17,18], we suspected an entropy increase by montelukast; however, there was reduced entropy as reflected by a cluster arising from central-frontal areas (*p* = 0.00459, electrode cluster corrected) and another cluster (*p* = 0.00459) extending in central-parietal regions (Figure 3A).

The permutation tests conducted for the EEG characteristics during the semantic memory task (WP-task) revealed no significant effects for the learning condition, however, during recall, a significantly reduced Hjorth activity (*p* = 0.0352) was observed, which was most pronounced in the occipital region (Figure 3B). For the episodic memory (VR) task, the EEG analysis revealed an effect on entropy. Here entropy was significantly reduced in frontal regions (*p* = 0.0078) but enhanced (*p* = 0.0078) at parietal areas. In further support, we found an acceleration of the brain-rate frontal (*p* = 0.0469) and enhancement of the Hjorth activity at central electrodes (*p* = 0.0313).

**Figure 4:**
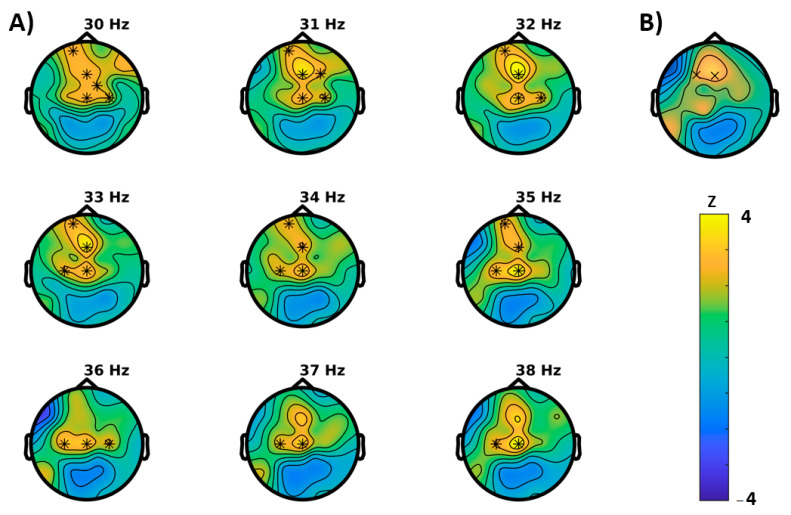
Results of the cluster based permutation tests for the congruent trials of the Simon task (BASELINE vs. MONTELUKAST, cluster corrected, x = *p* < 0.05, * = *p* < 0.01). (**A**) Cluster for spectral power and (**B**) Hjorth mobility.

**Figure 5:**
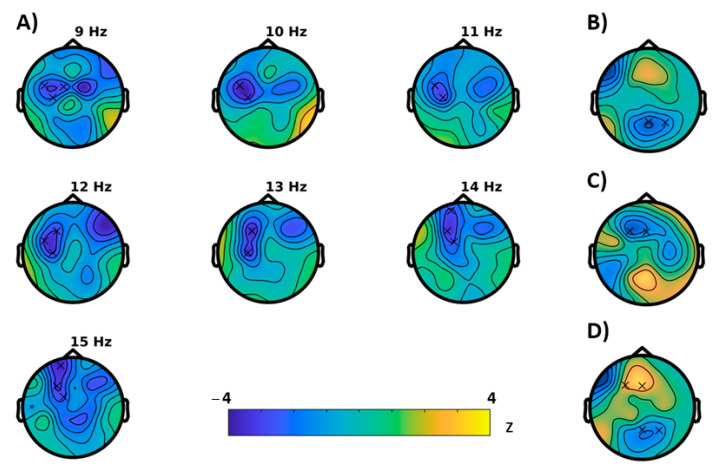
Results of the cluster based permutation tests for the incongruent trials of the Simon task (BASELINE vs MONTELUKAST, cluster corrected, x = *p* < 0.05). (**A**) Cluster for spectral power, (**B**) brain-rate, (**C**) entropy, (**D**) Hjorth mobility.

For the Simon task (visual attention), congruent and incongruent trials were analyzed separately. For congruent trials, permutation tests showed significant differences between baseline and follow-up for the following characteristics (Figure 4): An increase in power (*p* = 0.0039) that extended mainly in the gamma range and, in concordance, an increase of the Hjorth mobility (*p* = 0.0391) that can be interpreted as a shift in mean frequency to higher frequency ranges. In further agreement, both clusters were most likely to be expressed in frontocentral brain regions. For incongruent trials (Figure 5), instead, a reduction of power (*p* = 0.043, cluster-corrected) was observed in the alpha and beta frequency ranges. Like in the congruent trials, this effect most likely emerged from frontocentral brain regions. Furthermore, a decrease of the brain-rate (*p* = 0.043, cluster-corrected) was measured in postcentral-parietal regions, indicating a slowing of the central frequency, in agreement with a reduced Hjorth mobility respective mean frequency (*p* = 0.0352) in parietal regions as well. With extending clusters at frontal sites instead, the Hjorth mobility was elevated (*p* = 0.0352) and a reduction of entropy of the signal was revealed (*p* = 0.0117).

## 4. Discussion

The present study aimed to pilot the effects of montelukast on the human CNS. Twelve asthma patients were assessed for cognitive and electrophysiological effects before and after 8 weeks of treatment with montelukast with a sole indication as an asthma medication. Our data did not support findings that montelukast promotes cognitive functioning [13,14,17]. We did not observe any changes that could be attributed to montelukast according to neuropsychological measurements of memory, attention, and psychiatric symptoms. In the EEG we found modulations of different features during rest and cognitive stimulation, which could be interpreted as neurophysiological changes. However, these effects were divergent and did not correlate with cognitive performance.

Recent findings in rodents suggested the beneficial impact of montelukast on learning and memory, in aged rats [13] and in rats with dementia with Lewy bodies [14]. Both studies rely on the Morris water maze task, which tests for hippocampus-dependent (episodic) spatial memory and is an established task in behavioral rodent studies [53]. The extent to which the rodent memory system is comparable to that of humans is controversial; in order to provide comparable testing situations in humans it is today possible to implement egocentric and allocentric measures of spatial and episodic memory in a virtual reality environment [54]. Consequently, to mimic similar cognitive demands in our study we conducted a virtual reality paradigm [34] where participants had to recollect details after navigating through a virtual town. We additionally involved a semantic memory paradigm and a neuropsychological screening for verbal memory (VLMT) to account for possible effects on other memory domains. However, as we measured no performance changes in any memory task, we could not provide evidence for generalizability of the promising findings from rats to humans. Besides differences in cognitive demands, a possible explanation could be species-related differences in hippocampal neurogenesis [55] and its different impact on hippocampal memory formation.

In addition to memory, normal and pathological age-related deteriorations are reported for several cognitive functions, including deficits in attention, loss of inhibitory control and slowing of executive functions [6,56]. To account for modulation on these functions, we conducted the Simon task and TAP (attention and inhibition control) and the EpiTrack^®^ (executive and general neurological functioning). However, we did not observe any effect after 8 weeks montelukast treatment in these tasks. These results are at odds with a recent retrospective study comparing patients with previous montelukast prescriptions to patient groups on other medications. This study showed small effects for better performance of the montelukast group on several neuropsychological tests (e.g., finger tapping, digit-symbol coding, or subjective memory) [17]. Given the small effect sizes in their study, the authors argued that the prescribed dose of montelukast (10 mg/kg) might not have the ability to cross the blood-brain barrier. To this argument, we add that the duration of medication might be also a crucial factor. As there is no information for controlling for medication duration in their study, we suspect that mixing acute and chronic patients could contribute to their small effect sizes and explain the difference to our outcome, where we measured no cognitive effect at all but included a comparably short medication duration in relation to chronic asthma treatment.

Due to the heterogeneity of the effects in EEG biomarkers, these results are not straightforward to interpret. A considerable number of studies have shown the effects of normal ageing, cognitive decline and dementia on various EEG parameters [25,27,28,57,58,59,60]. In general, a slowing of the tonic EEG and a reduced complexity of the signal has been associated with ageing and pathology [30,31,32]. In the frequency domain, the dominant rhythm decreases with age and even more so in conditions with progressive cognitive decline [28,29]. Slowing of the dominant background rhythm and decrease in power of the upper alpha bound are reported to be a normal correlate of ageing [61] and increase of theta activity accompanied by decrease in higher frequency bands is even more pronounced in older individuals with mild cognitive impairment or Alzheimer’s dementia compared to healthy controls [62]. These changes of EEG background activity correlate negatively with cognitive performance [25,28,59,62]. In contrast, acceleration of the EEG background rhythm is a biomarker for the response to treatment of Alzheimer’s disease [19,63] and is associated with better cognitive performance [25]. We, therefore, hypothesized that Montelukast could accelerate the mean frequency and shift power to higher frequency bands and that such tonic changes could be detected during rest.

We further hypothesized that positive behavioral performance would be in line with these changes. However, we found no modulations in any frequency band or acceleration of the mean-frequency measures during the resting EEG.

During cognitive tasks, i.e., the ‘phasic’ or event-related EEG, the brain dynamically adapts to task-specific cognitive demands, which is reflected in temporal and local changes in brain activity [25]. How the event-related EEG is modulated by age or pathology is poorly understood and mainly assessed by ERD or ERP measures so far [23,25,38]. Therefore, during cognitive stimulation, the investigation of possible effects of montelukast for our extracted biomarkers remained exploratory. Here, we found an elevated mean frequency during the recall of the episodic memory paradigm and an increase in signal variance (Hjorth activity). Additionally, during the congruent trials of the attention and inhibition control task (Simon task) an elevated mean frequency and additionally an increase in gamma power was observed. In contrast, during incongruent trials of the Simon task, a reversed pattern was observed. Here, alpha and beta decreased, and the mean frequency was reduced. The differences in the EEG could be in the nature of the different demands of these two conditions and has already been reported for other EEG measures, e.g., event-related potentials [38] and ERDs [23]. Generally, as these differences were not reflected in the behavioral data, no conclusions can be drawn about quality, i.e., whether these changes would be beneficial for the treated individuals or not.

We further hypothesized that a possible positive effect of montelukast might be reflected in increased signal complexity, here measured by Shannon’s entropy. We found significant effects for entropy in almost all of our EEG conditions (except for rest 1). However, only for semantic memory retrieval were the results consistent with our hypothesis. It should be noted that complexity in (neural) systems is generally suggested to reflect the capacity of the system to process information [27]. In this sense, one could assume that a complex brain is a healthy brain. However, there is no single definition of complexity and there are various measures such as Shannon entropy, approximate entropy, sample entropy, etc., that give different results in the same data [64] and largely depend on temporal and spatial properties [27].

Overall, we found evidence of a possible effect of montelukast on the EEG. This is consistent with previous findings in rodents where effects on the EEG were measured in animal models of epilepsy [20,21]. The most consistent results were found for the episodic memory paradigm. However, the qualitative interpretation of our EEG results is difficult because we did not find behavioral correlates for our effect.

### Limitations

Besides the small sample size, there are several limitations of our study. Some of them may explain or contribute to the fact that we did not find any cognitive side effects. First of all, the most critical factor is that our cohort, which consisted of asthma patients but otherwise healthy subjects with a median age of 57.5 years, does not represent the elderly. Thus, it can be questioned whether any effects of montelukast can be expected in neurological and psychiatric healthy middle-aged individuals. So far, there is only evidence for effects in 20-month-old rats [13], which equals approximately 60 human years [16] and from [17], who also included only human individuals older than 60. Thus, a sample with 50% of the subjects younger than 58 years may therefore systematically underestimate possible effects.

Furthermore, there is little evidence that montelukast penetrates the human BBB in an efficient manner. Since we did not investigate the cerebrospinal fluid concentration of montelukast in this study, a conclusion from our EEG findings to a CNS efficacy of montelukast is problematic. In addition, such effects may be confounded by an improved lung function due to the asthma treatment, which we did not consider in this study. The tests were done by the treating physician, based on clinical grounds alone. Thus, we were unable to control for this potential confounder.

Another important factor why the translation from rodents to humans is difficult is the possible differences in the temporal dynamics of neurodegeneration and neurogenesis between species [55,65]. According to [13], the critical lever for montelukast-induced improvement in cognition is not anti-inflammatory activity, but the triggering of neurogenesis by stimulating proliferation of hippocampal progenitors. However, the comparability or scaling of neurogenesis processes from rodents to humans is not clear [54,64,65] neither its impact on cognition [66]. Thus, inhibition of the age-related depression of neurogenesis by montelukast may initiate the same but slower processes in humans and result in a later onset of efficacy. An 8 weeks treatment with 10 mg/kg montelukast per day like in our study may not lead to measurable cognitive effects in humans. In view of this and likely differences in pharmacokinetics, further studies in humans could investigate longer treatment periods, ideally by comparing different drug doses in a placebo-controlled trial.

In summary, this pilot study tested a potential approach to assessing the effects of montelukast on the CNS. We found no cognitive effects, but detected potential electrophysiological changes. These results must be viewed in light of the limitations of a small sample size, lack of blinding and lack of control for asthma improvement as a potential confounder. Nevertheless, this study may provide the impetus and basis for further, more advanced clinical trials that follow this approach and ideally account for this study’s limitations and include a control group.

## Figures and Tables

**Table 1:** Results of all behavioral testings (Neuropsychology and cognitive EEG).

Modality	Test	Scale	Mdn B	Mdn M	*z*	*p*	*r*
Adverse	AEP	Total score	36	35	−1.38	0.404	−0.28
event	EpiTrack	Interference	5.5	5	−0.71	0.728	−0.14
		Connect digits	5	5.5	−0.69	0.728	−0.14
		Connect letters	5	6	1.31	0.404	0.27
		Maze test	6	6	0.45	0.801	0.09
		Verbal fluency	5	6	2.11	0.197	0.43
		Digit span	5	5	1.63	0.394	0.33
		Total score	32	33	1.34	0.404	0.27
Memory	VR	Recollection	31.5	30.5	−0.59	0.755	−0.12
	WP	Recollection	21.5	19	−0.31	0.801	−0.06
	VLMT	Learning	52.5	58	1.57	0.394	0.32
		Consolidation	2	3.5	1.62	0.394	0.33
		Recognition	13.5	9.5	−0.62	0.755	−0.13
Attention	Simon	RT C × C	462	446	−0.31	0.801	−0.06
		RT IC × IC	510	506	−0.39	0.801	−0.08
		RT C × IC (B)	462	510	3.06	0.049 *	0.62
		RT C × IC (M)	446	506	2.98	0.049 *	0.61
		AC C × C	99	100	1.48	0.404	0.3
		AC IC × IC	96	98	1.26	0.419	0.26
		AC C × IC (B)	99	96	−2.5	0.106	−0.51
		AC C × IC (M)	100	98	−2.67	0.087	−0.54
	TAP	RT auditive	614	617	−0.39	0.801	−0.08
		RT visual	785	829	0.94	0.62	0.19
		RT tone (+)	242.5	233.5	1.33	0.404	0.27
		RT tone (−)	241	242.5	0.78	0.71	0.16
Mood	HADS	Anxiety	5	5	−0.77	0.71	−0.16
		Depression	2	1	−0.33	0.801	−0.07
Personality	BSI	Total score	60	64	2.12	0.197	0.43
	FPZ	Neuroticism	73.5	80.5	0.22	0.824	0.05
		HOPS	55.5	56.5	−0.22	0.824	−0.05
		Extraversion	61.5	60	−1.35	0.404	−0.28
		Addiction	14	16	1.63	0.394	0.33
		Delusion	5	4.5	0.96	0.62	0.2
		Total score	212	218.5	−0.43	0.801	−0.09

Notes: Wilcoxon singed rank tests were performed for all comparisons. All p-values were corrected using the Benjamini–Hochberg procedure. Indicator of significance: * *p* < 0.05. (*n* = 8), Cognitive EEG (*n* = 8), HADS *(n* = 11), all other tests with *n* = 12. All contrasts were calculated across baseline vs follow-up. Only for the Simon effect (C × IC) contrasts were perfomed within baseline (B) or within follow-up (M). Abreviations: Reaction time (RT), accuracy (AC), congruent trial (C), incongruent trial (IC), with alarm tone (+), without alarm tone (−).

## Data Availability

The data presented in this study are available on reasonable request from the corresponding author.

## Supplementary Materials

The following are available online at https://www.mdpi.com/article/10.3390/brainsci11050547/s1, Table S1: Results (*p*-values) of the cluster based permutation tests for all EEG conditions.
