# Supplementary material for: Cognitive Effects of Montelukast: A Pharmaco-EEG Study
Source: Brain Sci. 2021 Apr 27;11(5):547. doi: 10.3390/brainsci11050547 (PMC8145277; doi:10.3390/brainsci11050547)
Supplement: Supplementary file 1 [file brainsci-11-00547-s001.zip › brainsci-1165006-supplementary.pdf]

Supplementary File

Table S1.Results (p-values) of the cluster based permutation tests for all EEG conditions.

Condition	Power	Brain-rate	Entropy	Hjorth A	Hjorth M	Hjorth C
**Rest 1**	-	-	-	-	-	-
**Rest 2**	-	-	- 0.0459	-	-	-
	-	-	- 0.0459	-	-	-
**WP learn**	-	-	-	-	-	-
**WP recall**	-	-	-	- 0.0352	-	-
**VR learn**	-	+ 0.0469	+ 0.0078	+ 0.0313	-	-
	-	-	- 0.0078	-	-	-
**Simon C**	+ 0.0039 (γ)	-	-	-	+ 0.0391	-
**Simon IC**	- 0.0430 ($\alpha\beta$)	- 0.0430	- 0.0117	-	+ 0.0352	-
	-	-	-	-	- 0.0352	-

Notes: Cluster based permutation tests based on the t-statistics for dependent samples were performed across BASELINE vs MONTELUKAST. Level of significance, p = <.05. Resting EEG (n = 10, 1024 permutations), cognitive EEG (n = 8, 256 permutations), positive cluster (+), negative cluster (-). Frequency and electrode cluster correction was performed for power, electrode cluster correction for all other measures.
